# Roflumilast Powders for Chronic Obstructive Pulmonary Disease: Formulation Design and the Influence of Device, Inhalation Flow Rate, and Storage Relative Humidity on Aerosolization

**DOI:** 10.3390/pharmaceutics13081254

**Published:** 2021-08-13

**Authors:** Mohammad A. M. Momin, Bishal Raj Adhikari, Shubhra Sinha, Ian Larson, Shyamal C. Das

**Affiliations:** 1 School of Pharmacy, University of Otago, Dunedin 9054, New Zealand; mammomin@vcu.edu (M.A.M.M.); adhbi024@student.otago.ac.nz (B.R.A.); shubhra.sinha@otago.ac.nz (S.S.); 2 Department of Pharmaceutics, School of Pharmacy, Virginia Commonwealth University, Richmond, VA 23298, USA; 3 Drug Delivery, Disposition and Dynamics, Monash Institute of Pharmaceutical Sciences, Parkville Campus, Monash University, 381 Royal Parade, Parkville, VIC 3052, Australia; ian.larson@monash.edu

**Keywords:** roflumilast, L-leucine, trehalose, chronic obstructive pulmonary disease, dry powder

## Abstract

Roflumilast is currently administered orally to control acute exacerbations in chronic obstructive pulmonary disease (COPD). However, side effects such as gastrointestinal disturbance and weight loss have limited its application. This work aimed to develop an inhalable roflumilast formulation to reduce the dose and potentially circumvent the associated toxicity. Roflumilast was cospray-dried with trehalose and L-leucine with varied feed concentrations and spray-gas flow rates to produce the desired dry powder. A Next-Generation Impactor (NGI) was used to assess the aerosolization efficiency. In addition, different devices (Aerolizer, Rotahaler, and Handihaler) and flow rates were used to investigate their effects on the aerosolization efficiency. A cytotoxicity assay was also performed. The powders produced under optimized conditions were partially amorphous and had low moisture content. The powders showed good dispersibility, as evident by the high emitted dose (>88%) and fine particle fraction (>52%). At all flow rates (≥30 L/min), the Aerolizer offered the best aerosolization. The formulation exhibited stable aerosolization after storage at 25 °C/15% Relative Humidity (RH) for one month. Moreover, the formulation was non-toxic to alveolar basal epithelial cells. A potential inhalable roflumilast formulation including L-leucine and trehalose has been developed for the treatment of COPD. This study also suggests that the choice of device is crucial to achieve the desired aerosol performance.

## 1. Introduction

Chronic obstructive pulmonary disease (COPD) is a progressive airflow obstruction associated with chronic lung inflammation, which impairs a patient’s quality of life, and acute exacerbation can lead to death [1]. In contrast to other diseases, the morbidity and mortality rate of COPD continue to increase, and COPD is a major global health burden [2]. Bronchodilators, corticosteroids, muscarinic antagonists, methylxanthines, phosphodiesterase-4 (PDE4) inhibitors, and mucolytic agents currently constitute the treatment for COPD [3]. Among these, the PDE4 inhibitor shows particular promise against COPD as PDE enzymes hydrolyze and inactivate cyclic adenosine monophosphate (cAMP) and cyclic guanosine monophosphate (cGMP), which regulate smooth muscle relaxation and inflammatory mediator release [4].

Roflumilast (3-cyclo-propylmethoxy-4-difluoromethoxy-*N*-[3,5-dichloropyrid-4-yl]-benzamide) is a selective PDE4 inhibitor used to control acute exacerbations in patients with severe COPD [3]. Although the current roflumilast oral therapy has a clear purpose in the prevention of acute exacerbations, it comes with undesired adverse effects including gastrointestinal disturbances, insomnia, and weight loss [5,6]. As COPD is marked by local inflammation, a formulation delivering the drug directly to the site of action could circumvent the associated side effects by reducing systemic exposure. Inhaled roflumilast was more effective than oral roflumilast in curbing and relieving allergen-induced air-flow obstructions in Brownian Norway rats when intratracheally administered [7]. A spray-dried roflumilast dry powder formulation incorporating hydroxypropyl-β-cyclodextrin (HPβCD) has been reported [8]. While HPβCD has been shown to be non-toxic upon inhalation, it was found to slightly increase lymphocyte count in the bronchoalveolar lavage, the implications of which remain unknown [9]. The fine particle fraction (FPF) of the reported formulations was between 44 and 58% when dispersed from a Handihaler device at an inhalation flow rate of 60 L/min. A new formulation strategy could improve the FPF. In addition, the implications of using different devices and inspiratory flow rates on aerosol performance for such low-dose formulations have not been explored.

While formulations strategies are important for pulmonary drug delivery, the device used and the inspiratory flow rate are also of paramount importance [10,11,12]. The currently available dry powder inhaler (DPI) devices can broadly be categorized into low (<5 Mbar), medium (5–10 Mbar), and high-resistance (>10 Mbar) devices based on the pressure drop [11]. The Breezhaler and Aerolizer are examples of low-resistance devices. The Accuhaler, Clickhaler, Turbohaler, and Rotahaler are devices offering medium resistance, whereas high-resistance devices include the Handihaler and Easyhaler. Patients with COPD have compromised lung functions [11,13,14]. Therefore, an intricate balance between inspiratory flow rate and device resistance must be considered to achieve the desired aerosol performance.

The aim of this study was to develop a highly dispersible low-dose dry powder formulation of roflumilast using spray drying. For this, trehalose was selected to form the bulk of the formulation. Trehalose is a non-reducing sugar that is easily cleared by metabolism and is non-toxic to the lungs [15,16]. To achieve good dispersion, L-leucine was incorporated into the formulation. Other amino acids are also used as aerosolization enhancers, but L-leucine is the most widely used amino acid and is generally regarded as safe (GRAS) for inhalation delivery [15,17,18]. In addition to the formulation endeavors, the influence of device on aerosolization of the optimized formulation was also investigated. Three different devices (Aerolizer, Rotahaler, and Handihaler) were selected based on their resistance capacities. Moreover, the influence of the inhalation flow rate (30, 45, and 60 L/min) on aerosolization was examined. The cytotoxicity of the formulation on an alveolar cell line was also investigated.

## 2. Materials and Methods

### 2.1. Materials

Roflumilast and L-leucine were purchased from Hubei Yuancheng Technology Co. Ltd., Wuhan, China and Hangzhou Dayangchem Co., Ltd., Zhejiang, China, respectively. Acetonitrile, methanol, isopropanol (high-performance liquid chromatography, HPLC grade), and trehalose (analytical reagent, AR grade) were purchased from Merck, Germany. Ammonium acetate, glacial acetic acid (AR grade), and silicone oil (viscosity 10 cSt) were purchased from Sigma–Aldrich, St. Louis, MI, USA. Hard gelatin capsules (Size 3) were kindly donated by Capsugel Co., Ltd., Tokyo, Japan. Fresh Milli-Q water prepared using equipment from Millipore Corporation, Bedford, MA, USA was collected and filtered through 0.45 μm membrane filter manufactured by Phenomenex, CA, USA.

### 2.2. Quantification by HPLC Analysis

Roflumilast was analyzed using a validated reverse-phase HPLC method modified from Belal et al. [19]. A reverse-phase HPLC system (Shimadzu, Kyoto, Japan) equipped with an LC-20AD solvent delivery unit, Prominence photodiode array (PDA) detector (Shimadzu SPD-M20A), degasser (Shimadzu DGU-20A5), and autosampler operated with Class-VP 7.4SP4 software was used. The mobile phase, ammonium acetate buffer (pH 6.3), acetonitrile, and methanol (30:35:35% *v*/*v*) was pumped at a 1.0 mL/min flow rate through a Synergi Fusion RP80A C18 column (4 µm, 150 × 4.6 mm; Phenomenex, Torrance, CA, USA) connected to a C18 security guard (Phenomenex Fusion RP, CA, USA; 4.0 × 3.0 mm). An aliquot (20 µL) of each sample was injected at ambient temperature and detected at a wavelength of 251 nm with a total run time of 8 min. The calibration curve (0.25–80 µg/mL) was linear (*R*^2^ = 0.9999) with the limit of detection (LOD) and limit of quantitation (LOQ) of 0.04 and 0.11 µg/mL, respectively. The repeatability and reproducibility of the separately prepared quality control samples (10, 50, and 100 µg/mL) were within the acceptable limit (%Bias: ≤15; coefficient variation, %CV: ≤15).

### 2.3. Preparation of Powders, Study Design, and Optimization

A Buchi B-290 Mini Spray-Dryer (Buchi Labortechnik AG, Flawil, Switzerland) attached with a high-performance cyclone separator was used to produce the powder particles in a closed mode. Feed solutions were prepared in a co-solvent system of ethanol and water using nitrogen as an atomizing gas. The roflumilast was first dissolved in ethanol; trehalose and L-leucine were dissolved in water. Then, the two solutions were mixed and sonicated for 5 min to give the final desired feed concentration. Prepared feed solutions were spray-dried using the following constant variables: ratio of formulation components (roflumilast, L-leucine, and trehalose: 4, 24, and 72% *w*/*w*), co-solvent mixture (ethanol and water, 70:30% *v*/*v*), feed rate (2 mL/min), aspiration (100%), inlet temperature (165 °C), and nozzle diameter (0.7 mm) as well as independent variables: feed concentration and spray-gas flow rate (Table 1). The feed concentration and spray-gas flow rate were chosen as independent variables since these are the most prominent factors affecting the droplet formation, which eventually affects the spray drying yield and particle size. The outlet temperature was recorded based on the inlet temperature.

A $3^{2}$ full-factorial design study based on independent variables was undertaken to prepare the powder formulations (Table 2) and investigate the effect of the independent variables on particle size and process yield. The main effects of the factors on particle size and yield were analyzed by balanced ANOVA using Minitab^TM^ software (Minitab Inc., Version 16, State College, PA, USA). Triplicate batches of the optimized powder formulation based on particle size (with maximum process yield) analysis were prepared to evaluate the inter-batch variability in aerosolization and related properties. The roflumilast-only powder was prepared using the optimized spray-drying conditions after dissolving roflumilast in the ethanol and water co-solvent system at a total solid content of 0.75% *w*/*v*. The spray-dried powders from the sample collector were transferred into screw-capped glass vials and stored at room temperature in a desiccator until evaluation. The following equation was used to calculate the percentage yield of the spray-dried powders.

$$Yield\left(\%\right)=\frac{\mathrm{Mass}\mathrm{of}\mathrm{the}\mathrm{powder}\mathrm{obtained}\left(mg\right)}{\mathrm{Total}\mathrm{solid}\mathrm{content}\mathrm{of}\mathrm{each}\mathrm{formulation}\left(mg\right)}\times 100\%$$

### 2.4. Particle Size and Size Distribution by Laser Light Diffraction

The particle size distribution of the powders was determined using the LA-950 diffraction analyzer (Horiba Ltd., Kyoto, Japan). The samples were dispersed in isopropanol and transferred into a fraction cell for analysis. The refractive index of roflumilast (1.6) was used to measure the volumetric particle diameters. All experiments were done in triplicate for the optimized formulation. The span values were calculated using the following equation:

$$\mathrm{Span}\mathrm{value}=\frac{D90-D10}{D50}$$

where *D*90, *D*50, and *D*10 represent the equivalent diameters at 90%, 50%, and 10% cumulative volume of the particles, respectively.

### 2.5. Crystallinity by X-ray Powder Diffraction (XRPD)

The crystallinity of the powder samples was determined with an X’Pert PRO MPD PW3040/60 X-ray diffractometer (Malvern Panalytical, Malvern, UK) equipped with Cu Kα radiation. An aluminum holder was used to analyze the samples over a 2θ range of 5–35° at a rate of 2°/min. The PANalytical High Score software was used to analyze the diffractogram.

### 2.6. Residual Solvent Content by Thermogravimetric Analysis (TGA)

The residual solvent content of the formulations was determined using a Q50 thermogravimetric analyzer (TA Instruments, New Castle, DE, USA). Approximately 5 mg of each sample was placed in a platinum pan and heated at a rate of 10 °C/min from room temperature (≈25 °C) to 150 °C under a nitrogen purge. The weight change was analyzed using the TRIOS software (TA Instruments, New Castle, DE, USA).

### 2.7. Phase Transition by Differential Scanning Calorimetry (DSC) 

The thermal behavior and phase transition of the supplied and spray-dried powders were investigated using a Q100 DSC system (TA Instruments, New Castle, DE, USA) calibrated with indium. Approximately 5 mg of powder sample was placed into an aluminum DSC pan and sealed with an aluminum lid (TA instruments) using a manual pan crimper press (PerkinElmer, Boston, MA, USA). An empty aluminum pan sealed with a lid was used as a reference pan. The DSC pans were scanned at 5 °C/min over the range of 0 to 350 °C. During heating, nitrogen was used as a purging gas (at 50 mL/min).

### 2.8. Powder Density and Flow Property

A 1 mL tuberculin syringe was used to determine the powder density. The bulk volume was measured after adding powder into the syringe up to 0.5 mL mark, and the weight of the powder sample was used to calculate the bulk density before tapping. Approximately after 100 taps/min (until no volume change), the volume was observed (tapped volume) and used to calculate the tapped density from powder volume. Both bulk and tapped densities were used to calculate the flow property of the powders using the following equation:

$$Carr^{\prime }s\mathrm{Index}\left(\%\right)=\frac{\mathrm{Tapped}\mathrm{density}-\mathrm{Bulk}\mathrm{density}}{\mathrm{Tapped}\mathrm{density}}\times 100\%.$$

### 2.9. Particle Morphology by Scanning Electron Microscopy (SEM)

The surface morphologies of the powders were examined using a JEOL 6700F scanning electron microscope (JEOL Ltd., Tokyo, Japan). A K575X sputter coater (EM Technologies Ltd., Kent, England) was used to sputter-coat the particles on carbon sticky tape with a gold/palladium alloy. The images were generated at 5 kV acceleration voltage.

### 2.10. In Vitro Aerosolization Performance by Next-Generation Impactor (NGI)

The Next-Generation Impactor (NGI) from Copley Scientific Ltd., Nottingham, UK, was used to determine the in vitro aerosolization performances of the spray-dried powder formulations. A Copley TPK 2000 critical flow controller (Copley Scientific Ltd., Nottingham, UK) and an electronic digital flow meter (Copley Scientific Model DFM2000) were used to achieve the desired flow rate. Approximately 20 mg of the powder was filled into gelatin capsules (Capsugel Japan Inc., Kanagawa, Japan) and dispersed using an Aerolizer at a flow rate of 100 L/min over 2.4 s to inspire 4 L air. This volume (4 L) is considered as the normal forced inhalation capacity of an average-sized male. This 100 L/min flow rate was chosen since we used Aerolizer, which is a low-resistance device (pressure drop < 5 Mbar) [20]. Approximately 15 mL of water was placed in the pre-separator of the device, whereas the stages 1 to 7 (S1 to S7) and Micro-Orifice Collector (MOC) were coated with a layer of silicone oil (viscosity = 10^−5^ m^2^/s at 25 °C). The cut-off diameters for NGI stages 2–7 at 100 L/min flow rate were 3.42, 2.18, 1.31, 0.72, 0.40, and 0.24 μm, respectively. Following actuation, the dispersed powders retained on the device along with the capsule and the powder deposited in all the parts of NGI were collected using acetonitrile and methanol mixture at a ratio of 10:90% *v*/*v* and analyzed by the HPLC method. Triplicate samples were prepared for the in vitro aerosolization performances of each formulation. All the aerosolization performances were conducted at room conditions (20 ± 2 °C temperature and 32 ± 2% relative humidity (RH)).

The aerodynamic parameters (recovered dose, emitted dose (ED), fine particle dose (FPD), fine particle fraction (FPF), mass median aerodynamic diameter (MMAD), and geometric standard deviation (GSD) were calculated for each run. The recovered dose (RD) was the total amount of drug retained on the device and deposited in different stages of the NGI, including MOC. The ED was the RD minus drug retained on the device. The FPD was the amount of drug deposited in stages from S2 to MOC. The FPF (%) was FPD expressed relative to ED. %ED was expressed relative to RD. The MMAD and GSD were calculated using the CITDAS software (Copley Scientific Ltd., Nottingham, UK).

### 2.11. Stability Studies

A single batch of roflumilast-only and optimized cospray-dried powder was stored in an open Petri dish at two different RH conditions (15% and 75%) at 25 ± 2 °C for one month to investigate the effect of low and high humidity on aerosolization performance. After storage, the aerosolization performance and other physicochemical properties (morphology, crystallinity, and drug content) of the powders were evaluated by the methods mentioned above.

### 2.12. Effect of Inhaler Device and Flow Rate on Aerosolization

The influence of inhaler devices and airflow rates on the aerosolization performances of the powder formulations were examined using three different inhaler devices (Aerolizer, Rotahaler, and Handihaler) at three different flow rates (30, 45, and 60 L/min). Devices were selected based on their resistance (Aerolizer (pressure drop < 5 Mbar), Rotahaler (pressure drop 5–10 Mbar), and Handihaler (pressure drop > 10 Mbar) to airflow. Aerolizer (Novartis Pharmaceuticals UK Ltd., London, UK), Rotahaler (GlaxoSmithKline, London, UK), and Handihaler (Boehringer-Ingelheim, Ingelheim am Rhein, Germany) are low, medium, and high resistance devices, respectively [10,21]. The in vitro aerosolization was performed following the same procedure mentioned above. Triplicate experiments were carried out for each device at each flow rate.

### 2.13. Cytotoxicity Studies by MTT Assay

The cytotoxicity of the roflumilast-only and cospray-dried roflumilast formulations was conducted using MTT assay on A549 (alveolar basal epithelium) cell line (ATCC, MD, USA). The A549 cell line was cultured in F12K medium. The medium was supplemented with 10% (*v*/*v*) fetal bovine serum and 1% penicillin solution. The cells were incubated in 5% carbon dioxide and 95% relative humidity at 37 °C. Further seeding was done in microtiter plates at a density of 5 × 104 cells/well and allowed to stand overnight for cell adhesion. Then, the cells were treated with 0.5, 1, and 4 µg/mL of roflumilast-only with a final volume of 200 µL (*n* = 3 at each concentration) for each cell line. Similarly, 12.5, 25, and 100 µg/mL of cospray-dried formulation was used to be equivalent to the roflumilast-only powder concentrations of 0.5, 1, and 4 µg/mL, respectively. The concentrations were chosen based on low aqueous solubility (≈5 µg/mL) of roflumilast [22]. The cell viability (cytotoxicity) was calculated using the following formula:

$$\mathrm{Cell}\mathrm{vaibility}\left(\%\right)=\frac{\mathrm{Average}\mathrm{absorbance}\mathrm{by}\mathrm{treated}\mathrm{cells}}{\mathrm{Absorbance}\mathrm{by}\mathrm{control}\mathrm{cells}}\times 100\%.$$

### 2.14. Statistical Analysis

All data were expressed as mean ± standard deviation. The inter-batch variability and intra-batch variability for aerosolization were compared using the nested ANOVA. Subsequent statistical analyses were performed by one-way analysis of variance (ANOVA) with the Student–Newman–Keuls test (compare all pairs) as a post hoc test at (*p* < 0.05). Instat Graphpad Prism software (version 4.00; GraphPad Software, San Diego, CA, USA) and Minitab (version 16; Minitab Inc., State College, PA, USA) were used for statistical analysis. Mean, standard deviation, and percentage relative standard deviation (%RSD) were calculated using MS Excel.

## 3. Results and Discussions

### 3.1. Powder Production and Optimization

**Figure 1:**
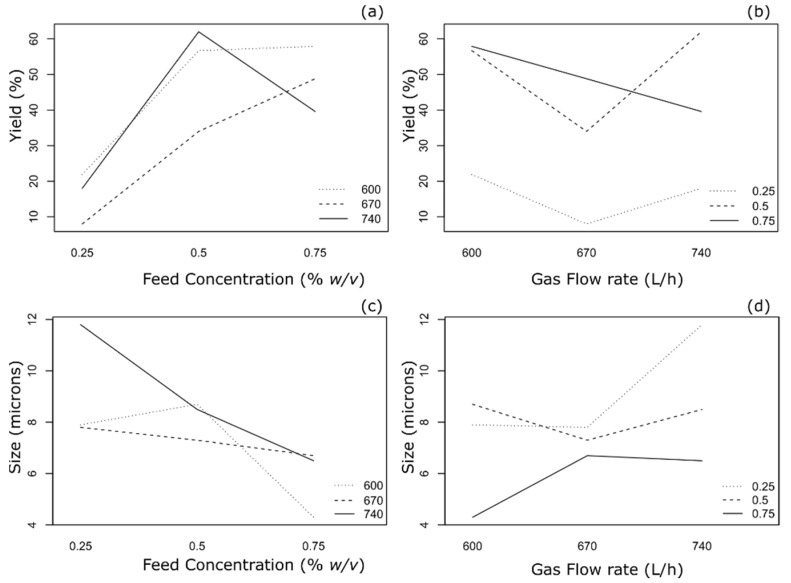
Main effects plots. (**a**) Effect of feed concentration on yield in different spray-gas flow rates. (**b**) Effect of spray-gas flow rate on yield in different feed concentrations. (**c**) Effect of feed concentration on particle size in different spray-gas flow rates. (**d**) Effect of spray-gas flow rate on particle size in different feed concentrations.

Nine formulations (F1 to F9) were spray-dried and investigated for the optimization of process yield and volumetric particle diameter. These parameters were chosen to achieve the target of producing inhalable size particles with maximum powder yield. In general, an increase in feed concentration increased the process yield, whereas the spray-drying gas flow rate did not have any effect on the yield (Figure 1, Table 3). Similarly, particle size was influenced only by feed concentration. With linear regression, although the effect of feed concentration was found to be significant (*p* < 0.05), the fit was poor (*R*^2^ = 0.58), suggesting the possibility of a non-linear relationship between the parameters.

Among the formulations, F5 exhibited satisfactory attributes. While having a relatively good yield, the formulation offered a D50 value of 4.3 µm, which is well within the desired range (1–5 µm) for deep lung delivery [23,24]. In addition, the low span value indicated a narrow particle size distribution of the spray-dried powders (Table 3).

Hence, the spray-drying conditions (feed concentration: 0.75% *w*/*v*, spray-gas flow rate: 601 L/h) of the F5 formulation were used for further particle preparation. Three batches of the cospray-dried powder and a single batch of roflumilast-only powder were produced using the optimized spray-drying conditions to further evaluate the in vitro aerosolization performance and physicochemical properties. The roflumilast-only powder was prepared and compared with the optimized cospray-dried powder to investigate how the optimized spray-drying conditions affect the powder properties and aerosol performance, since the aim of this study was to produce highly dispersible roflumilast powder for inhalation using spray drying.

### 3.2. Process Yield, Particle Size, and Drug Content of the Optimized Powder

The process yield of the optimized powder (F5) was >62.0%, whereas that of roflumilast-only powder was 47.7% (Table 4). The reason for the low process yield of roflumilast-only powder could be due to more powder deposition to the walls of the spray-dryer, which was apparent during the spray-drying process. The D50 values of both the powders were within the range for inhalation and reproducible. In addition, the drug content of the optimized powder formulations was within the acceptable range (75–125%) for inhalation preparations specified in the British Pharmacopoeia [25].

### 3.3. Crystallinity

**Figure 2:**
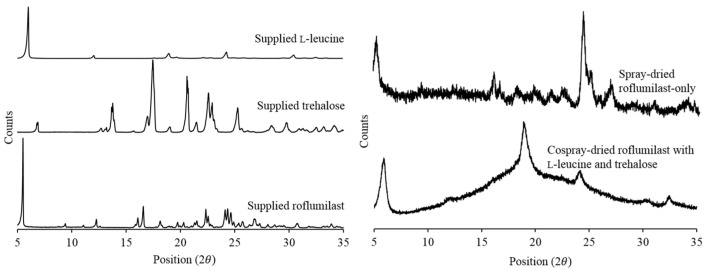
X-ray diffractograms of supplied and spray-dried powders.

The supplied roflumilast, L-leucine, and trehalose were crystalline, as confirmed by X-ray diffractograms (Figure 2). After spray-drying, the roflumilast-only powder still exhibited crystallinity; the lower intensity compared to the supplied material might have been due to the differences in particle size of these materials. The cospray-dried roflumilast with L-leucine and trehalose had diffraction peaks corresponding to those of L-leucine. The absence of peaks corresponding to crystalline roflumilast and trehalose peaks suggested the amorphous nature of these drugs in the cospray-dried powders. Trehalose was reported to be in amorphous state upon spray drying [26]. Similarly, it has been reported that L-leucine exists in a crystalline form after spray drying, although the intensity of crystalline peaks differs in the spray-dried L-leucine than the supplied L-leucine [17].

### 3.4. Moisture Content

The optimized cospray-dried formulations had the same moisture content as the spray-dried roflumilast-only powder (Table 4). There was no significant difference (*p* > 0.05) between the moisture content of batch 1 optimized cospray-dried powder (1.9 ± 0.7% *w*/*w*) and the other two batches (1.4 ± 0.1% *w*/*w*). In general, low moisture is desired in the spray-dried powders as a high amount of moisture can decrease particle dispersion by the formation of inter-particulate capillary forces [27].

### 3.5. Differential Scanning Calorimetry (DSC)

**Figure 3:**
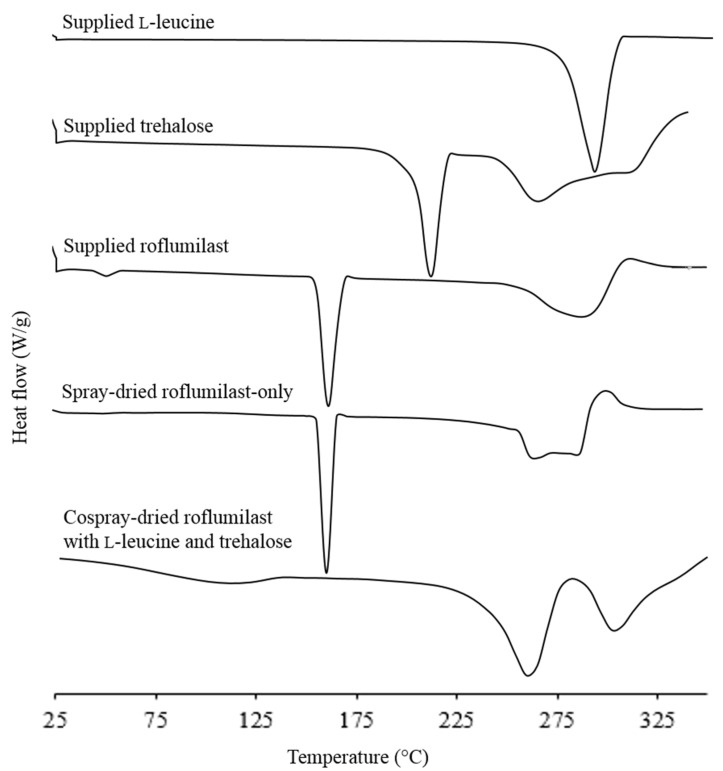
Differential scanning calorimetry (DSC) thermograms of supplied and spray-dried powders.

The DSC thermograms of supplied L-leucine and trehalose showed endothermic peaks respectively at 292 and 211 °C, corresponding to their melting points (Figure 3). Both supplied and spray-dried roflumilast had a melting point at 160 °C suggesting the crystallinity of the powders. The events higher than 225 °C possibly corresponded to thermal degradation as weight loss was seen with thermogravimetric analysis. When roflumilast was cospray-dried with L-leucine and trehalose, the endothermic peaks corresponding to the melting of roflumilast and trehalose at 160 and 211 °C could not be observed, suggesting the amorphous state of both roflumilast and trehalose in the cospray-dried powder. However, as the melting of L-leucine overlaid with possible degradation of roflumilast and trehalose, the melting peak of L-leucine could not be confirmed from the DSC thermogram.

### 3.6. Powder Density

The densities (bulk and tapped density) of the cospray-dried powders were lower than the spray-dried roflumilast-only powder (*p* < 0.05) (Table 4). However, there were no significant differences between the bulk and tapped densities of the three cospray-dried batches (*p* > 0.05), indicating the reproducibility of the spray-drying process. The average Carr’s index value of the spray-dried powders was (≈30), which is an acceptable flow property that is generally desired for ease during the capsule filling process [28].

### 3.7. Surface Morphology

**Figure 4:**
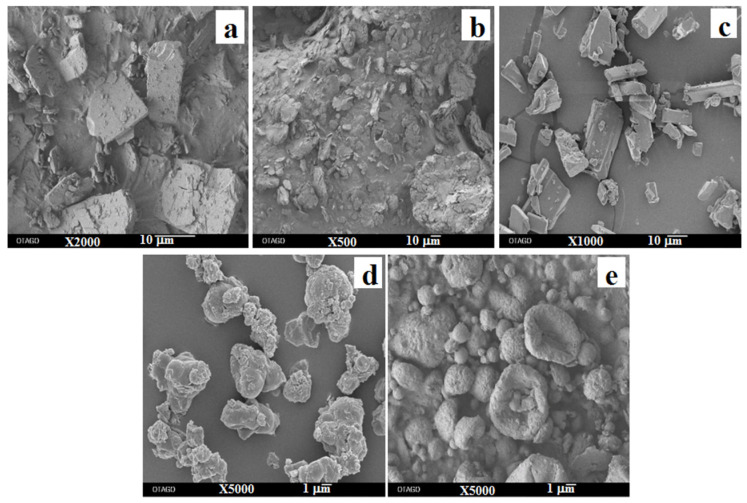
Representative scanning electron micrographs of: (**a**) supplied roflumilast; (**b**) supplied L-leucine; (**c**) supplied trehalose; (**d**) spray-dried roflumilast-only; and (**e**) spray-dried roflumilast with L-leucine and trehalose powders.

Figure 4 shows the SEM images of supplied materials and spray-dried powders. The supplied roflumilast and trehalose particles were irregularly angular shaped (Figure 4a,c), whereas the supplied L-leucine particles were flake shaped with rough surfaces (Figure 4b). The geometrical diameters of the supplied materials were >10 µm. After spray drying, the roflumilast-only particles were irregularly shaped and agglomerated (Figure 4d). However, cospray-dried roflumilast with L-leucine and trehalose particles were disc-shaped with rough surfaces (Figure 4e). The morphology and rough surfaces of the cospray-dried roflumilast particles might be due to the surface-active property of L-leucine, which influences droplet formation during atomization as well as surface attributes during the spray-drying process [29].

### 3.8. In Vitro Aerosolization Performance

**Figure 5:**
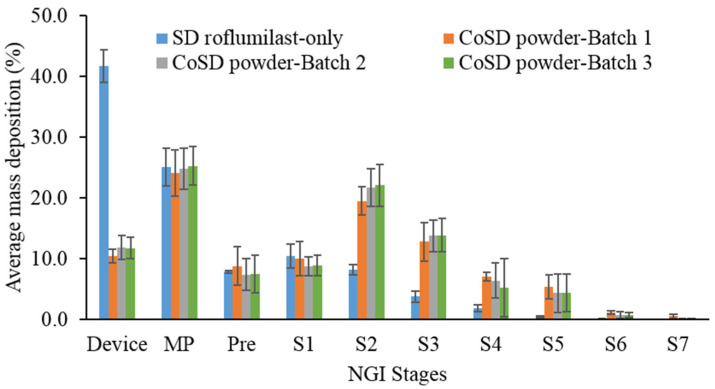
Drug deposition behavior of spray-dried (SD) and cospray-dried (CoSD) roflumilast powders on different stages of NGI (MP: Mouthpiece, Pre: Pre-separator, S1–S7 represents stages 1 to 7; error bars represent standard deviations, *n* = 3).

The aerosolization behaviors of the spray-dried roflumilast-only and cospray-dried powders have been summarized in Table 5. The roflumilast-only powder had lower aerosolization (ED and FPF) than the cospray-dried powders (*p* < 0.05). The majority of the powder was retained in the Aerolizer device for the roflumilast-only formulation (Figure 5) while the particle deposition in stages 2 to 7 (FPF) was only 25%. The low drug deposition in the lower parts of NGI for the formulation might be due to the larger aerodynamic diameter (Table 5) and agglomeration of roflumilast-only powder particles (Figure 4). In contrast, the cospray-dried powders had high emitted doses (>88%) and fine particle fractions (>52.0%) with no significant differences between the batches (*p* > 0.05). The higher ED of the cospray-dried powder could be due to the smaller aerodynamic diameter, lower density, and different particle morphology of these powder particles (Table 4 and Table 5, Figure 3). The MMAD values of around 3.5 µm and GSD between 1.6 and 2.0 µm indicated that the cospray-dried formulations were appropriate for deep lung delivery and had unimodal size distribution [30].

Hence, the cospray-dried formulation offered superior aerosol performance compared to the drug-only spray-dried powder, as evident by the ED and FPF.

### 3.9. Stability Studies

As inter-batch variability in the cospray-dried powder was not evident (*p* > 0.05) (Table 5), a stability study was conducted using a single batch. A comparison was made between the drug-only formulation and the cospray-dried formulation.

**Figure 6:**
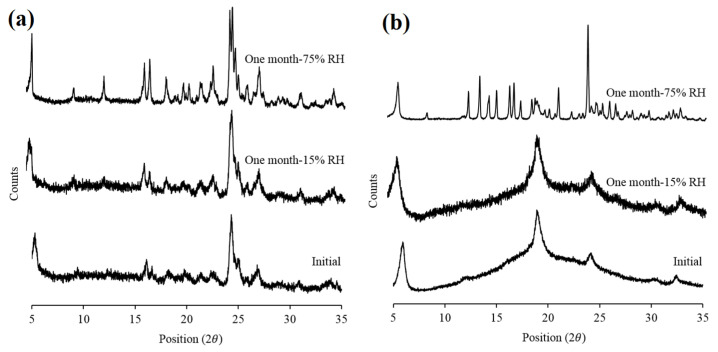
X-ray diffractograms of the spray-dried roflumilast-only (**a**) and cospray-dried (**b**) powders after one month storage at different storage conditions (for ease of comparison, the initial data from Figure 2 are shown here.).

**Figure 7:**
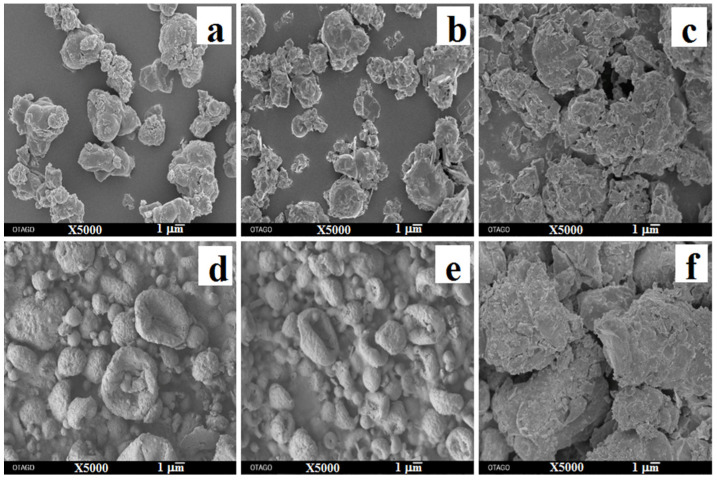
Scanning electron micrographs of spray-dried roflumilast-only powder after one-month storage at 15% RH (**b**) and 75% RH (**c**) and cospray-dried powder after one-month storage at 15% RH (**e**) and 75% RH (**f**) (For ease of comparison, initial SEMs from Figure 4 for roflumilast (**a**) and cospray-dried powder (**d**) are shown here).

At 25 °C/15% RH, no change in partial amorphicity and particle morphology was observed for any formulation suggested by XRD (Figure 6) and SEM images (Figure 7), respectively. The FPF remained unchanged for both the formulations over a month (*p* > 0.05) (Table 6), although an increase in ED was observed for the roflumilast-only formulation. The increase in ED might be likely due to the decay of electrostatic charge upon storage [31]. 

**Figure 8:**
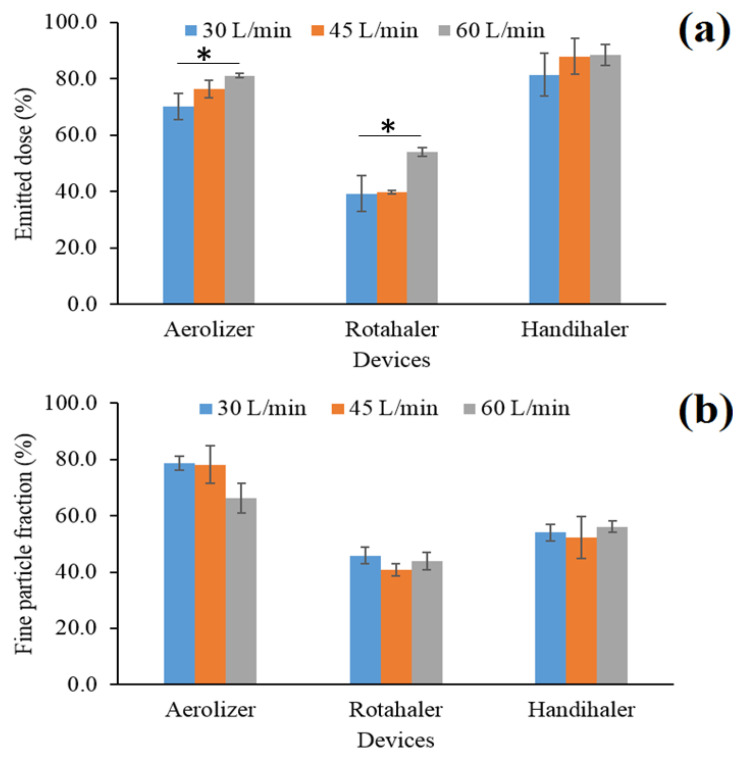
In vitro aerosolization of cospray-dried roflumilast powder using different devices at different flow rates: (**a**) emitted dose and (**b**) fine particle fraction (error bars are the standard deviations, *n* = 3). * The values are significantly different (*p* < 0.05).

At 25 °C/75% RH, the FPF of both the formulations decreased (*p* < 0.05); this decrease was mediated by moisture that resulted in particle fusion as evident in the SEM images (Figure 7). The co-spray dried formulation still offered better aerosolization at the end of the study compared to the roflumilast-only formulation, although the crystallization of all the excipients was observed for the co-spray dried formulation (Figure 8), suggesting that the co-spray dried particles could likely offer an advantage in terms of aerosolization stability. As at 15% RH, an increase in ED was observed for roflumilast-only powder, which is possibly due to electrostatic charge decay as well. No additional peaks were observed in the HPLC analysis, indicating the chemical stability of the drug during storage.

### 3.10. Effects of Device and Flow Rate on In Vitro Aerosolization

Following the preparation of roflumilast dry powder formulation with desired attributes, the effect of different devices and flow rates on aerosolization of cospray-dried powder was investigated. With Aerolizer, a low-resistance device (pressure drop < 5 Mbar), ED increased with an increase in flow rate, and the FPF decreased (Figure 8). As the FPF was calculated relative to ED, the decrease in FPF suggested that the FPD remained consistent in all flow rates and the mass corresponding to the higher ED in higher flow rates was deposited in the mouth piece, induction port, and S1. With Rotahaler, a medium-resistance device (pressure drop 5–10 Mbar), ED increased with an increase in flow rate, but no significant difference was observed in FPF (*p* > 0.05). The ED between the flow rate of 30 and 45 L/min was not significant (*p* > 0.05). With Handihaler, a high-resistance device (pressure drop > 10 Mbar), no change in ED (*p* > 0.05) or FPF (*p* > 0.05) was observed with a change in flow rate.

In general, for all the devices, there is a trend of increase in ED with an increase in flow rate; however, such a trend was not observed in case of FPF. Aerolizer and Handihaler showed higher ED, but the FPF of Aerolizer was higher compared to the FPF of the other two, suggesting that ED depended upon both device type and flow rate, but FPF was mainly dependent upon device, potentially independent of the flow rate. Among different resistance devices, dispersion of the dry powder depends mainly on flow rate for low-resistance devices, but the dependency tends to decrease with an increase in resistance [11]. Consistent with similar observations, a measure of dispersion (ED) was found to be relatively dependent on the flow rate for low (Aerolizer) and medium (Rotahaler) resistance devices but not for the high-resistance device (Handihaler). However, in all flow rates, FPF was higher in the low-resistance device (Aerolizer) than the medium (Rotahaler) and high-resistance (Handihaler) devices. This finding contradicts with the general understanding of increase in ED and FPF of dry powder inhalers with the increase in device resistance (i.e., increase in pressure drop) and flow rates. However, there are some exceptions of this rule, where ED and FPF can decrease with an increase in pressure drop and flow rates that are more related to formulations and device type, which may be applicable in our case [32]. The differences in the in vitro aerosol performances of the devices could be due to the differences in the dispersion mechanisms of the used devices. Although all the devices were capsule-based DPI devices, there was a difference in capsule piercing before actuation, capsule movement during actuation, etc. which might have contributed to the differences in powder dispersion and eventually deposition in the lungs (lower parts of NGI). (As COPD patients usually have compromised lung function, checking the inspiratory flow rate using spirometry measurements is important in achieving the desired deep lung delivery [12]. Here, the study suggests that the cospray-dried formulation is likely to achieve consistent FPF of >52% with Aerolizer when the inspiratory flow rate is as low as 30 L/min.

### 3.11. Cytotoxicity

**Figure 9:**
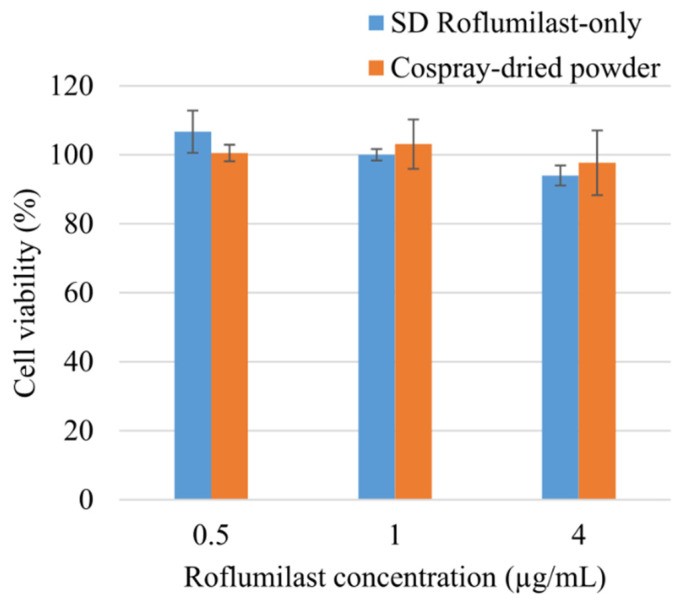
Cytotoxicity studies on A549 cells using roflumilast-only and cospray-dried powder (error bars are means ± standard deviations, *n* = 3).

The cytotoxicity profile of spray-dried roflumilast-only and cospray-dried powders was assessed using the A549 cell line, as shown in Figure 9. The cospray-dried formulation did not show toxicity on A549 cells up to 100 µg/mL (roflumilast concentration—4 µg/mL). The cell viabilities in both roflumilast and the formulation were above 94% in the concentrations tested, suggesting that the excipients used alongside roflumilast did not have any cytotoxic effect on the alveolar basal epithelium.

## 4. Conclusions

A potential inhalable roflumilast dry powder formulation to control acute exacerbations in patients with severe COPD can be developed by cospray-drying roflumilast with L-leucine and trehalose. While formulations endeavors are undertaken to achieve deep lung delivery, the importance of the choice of the device should not be overlooked, as lung function may be compromised in COPD patients, and the minimum inspiratory flow rate required for consistent drug delivery can vary with the DPI device being used for delivery.

## Figures and Tables

**Table 1:** Independent variables used in the factorial study.

Factors	Levels		
	Low (1)	Medium (2)	High (3)
A: Feed concentration (% *w*/*v*)	0.25	0.50	0.75
B: Spray-gas flow rate (L/h)	601	670	742

**Table 2:** The 3^2^ full-factorial design for the preparation of powder formulations.

Formulation	Level of Factor in the Experiment	
	A	B
F1	1	1
F2	2	1
F3	2	2
F4	2	3
F5	3	1
F6	3	2
F7	1	2
F8	3	3
F9	1	3

**Table 3:** Cospray-dried powders of roflumilast (F1 to F9) with L-leucine and trehalose, their spray-drying yield, and particle size distribution.

Formulation	Process Yield (%)	Volumetric Particle Diameter			
		D10 (µm)	D50 (µm)	D90 (µm)	Span
F1	21.9	4.8	7.9	13.7	1.1
F2	56.7	7.2	8.7	10.4	0.4
F3	34.0	3.3	7.3	13.3	1.4
F4	62.0	5.3	8.5	13.2	0.9
F5	57.9	1.9	4.3	8.9	1.6
F6	48.8	4.2	6.7	10.1	0.9
F7	8.0	3.4	7.8	12.6	1.2
F8	39.6	5.5	6.5	7.6	0.3
F9	18.0	6.5	11.8	18	1.0

**Table 4:** Process yield, particle size, moisture content, and flow property of spray-dried (SD) and cospray-dried (CoSD) roflumilast powders produced using the optimized spray-drying conditions. Data are means ± standard deviations, *n* = 3; yield, bulk density, tapped density, and Carr’s index values are from the single determination of each batch.

Formulation	Yield (%)	Particle Size (D50)	Moisture Content (%)	Bulk Density (g/mL)	Tapped Density (g/mL)	Carr’s Index (%)
SD roflumilast-only	47.7	5.5 ± 0.4	1.1 ± 0.2	0.363	0.539	32.7
CoSD powder-Batch 1	62.3	4.9 ± 0.3	1.9 ± 0.7	0.345	0.489	29.6
CoSD powder-Batch 2	62.5	5.4 ± 0.2	1.4 ± 0.1	0.356	0.495	28.1
CoSD powder-Batch 3	64.7	5.2 ± 0.1	1.4 ± 0.1	0.352	0.514	31.5

**Table 5:** In vitro aerosolization (emitted dose (ED), fine particle fraction (FPF)) and aerodynamic properties (mass median aerodynamic diameter (MMAD), geometric standard deviation (GSD)) of spray-dried (SD) and cospray-dried (CoSD) roflumilast powders.

Formulation	ED (%)	FPF (%)	MMAD (µm)	GSD
SD roflumilast-only	58.3 ± 2.7	25.5 ± 5.3	5.3 ± 0.7	2.0 ± 0.0
CoSD powder—Batch 1	89.6 ± 1.1	52.0 ± 5.4	3.5 ± 0.2	2.0 ± 0.3
CoSD powder—Batch 2	88.1 ± 2.0	53.5 ± 4.5	3.6 ± 0.2	1.7 ± 0.0
CoSD powder—Batch 3	88.2 ± 1.8	52.7 ± 5.3	3.7 ± 0.3	1.6 ± 0.1

**Table 6:** In vitro aerosolization of spray-dried and cospray-dried roflumilast powders after one month of storage at 15% and 75% RH and 25 °C. SD and CoSD mean spray-dried and cospray-dried, respectively; ED: emitted dose; FPF: fine particle fraction; data are means ± standard deviations, *n* = 3; initial data from Table 5 are used for comparison. * The value is significantly different (*p* < 0.05) to the value on day 0 (initial).

Formulation	ED (%)			FPF (%)		
	Initial	15% RH	75% RH	Initial	15% RH	75% RH
SD roflumilast-only	58.3 ± 2.7	89.5 ± 0.8 *	85.8 ± 1.1 *	25.5 ± 5.3	23.3 ± 0.3	14.1 ± 0.4 *
CoSD powder	88.1 ± 2.0	87.9 ± 2.1	85.1 ± 1.2	53.5 ± 4.5	59.0 ± 1.2	31.3 ± 1.5 *

## Data Availability

Data is contained within the article.
